# Supplementary material for: Cell line identity rather than medium composition determines transcriptomic profiles of HepaRG and HuH7 cells cultured in chemically defined or serum-based media: comparison with primary human hepatocytes
Source: Arch Toxicol. 2026 Jul 14;100(12):5481–96. doi: 10.1007/s00204-026-04502-z (PMC13748311; doi:10.1007/s00204-026-04502-z)
Supplement: Supplementary file 2 [file 204_2026_4502_MOESM7_ESM.docx]

Cell line identity rather than medium composition determines transcriptomic profiles of HepaRG and HuH7 cells cultured in chemically defined or serum-based media: comparison with primary human hepatocytes

Ahmed S. M. Ali^1^, Heike Sprenger^2^, Albert Braeuning^2^, Jens Kurreck^1*^

^1^ Technische Universität Berlin, Chair of Applied Biochemistry, Berlin, Germany

^2^ German Federal Institute for Risk Assessment (BfR), Berlin, Department Chemical and Product Safety, Germany

***Corresponding author:**

Prof. Dr. Jens Kurreck

Technische Universität Berlin, Institute of Biotechnology

Department of Applied Biochemistry, TIB 4/3-2

Gustav-Meyer-Allee 25, 13355 Berlin, Germany

Email: [jens.kurreck@tu-berlin.de](mailto:jens.kurreck@tu-berlin.de)

**SM1. HeLa cell culture and RNA isolation**

HeLa cells were cultured under two parallel conditions (serum-supplemented medium versus chemically defined medium, CDM) prior to RNA isolation. For serum-supplemented culture, cells were thawed from a cryopreserved FBS-containing stock and maintained in DMEM (low glucose) (L0064, Biowest) supplemented with FBS (10%; S-10-L, c.c.pro GmbH), L-glutamine (2 mM; X0550, Biowest), and non-essential amino acids (NEAA; 1%; X0557, Biowest). For CDM culture, cells were cultured in CDM developed by Nessar et al. based on DMEM/F12 (L0090, Biowest) supplemented with NEAA (1%; X0557–100, Biowest), HEPES (15 mM; L0180, Biowest), D-glucose (0.1% w/v; G8769, Sigma-Aldrich), L-glutamine (2 mM; X0550, Biowest), insulin–transferrin–selenium (ITS; 1×; 41400045, Gibco), recombinant human epidermal growth factor (hEGF; 100 ng/mL; PHG0313, Sigma-Aldrich), and hydrocortisone (10 µg/mL; sc-250130, Santa Cruz Biotechnology) (Nessar et al. 2025). Cells were maintained for five consecutive passages in triplicate in T25 flasks (TPP) under standard culture conditions (37 °C, humidified atmosphere, 5% CO₂).

Total RNA was isolated using the same workflow described for HepaRG and HuH7 cells. Briefly, at ~90% confluence, monolayers were washed with phosphate-buffered saline (PBS) to remove residual medium and lysed directly in RLT buffer supplemented with β-mercaptoethanol (1% v/v), using 700 µL lysis buffer per T25 flask. RNA was purified using the QIAwave RNA Kit (50) (74534, Qiagen) according to the manufacturer’s instructions, and RNA concentration and purity were assessed using a NanoDrop 2000 UV-Vis spectrophotometer (Thermo Scientific).

**Table S1**. Composition of the chemically defined media used for HepaRG cells.

| **Component** | **Final concentration** | **Supplier** | **Catalog number** |
| --- | --- | --- | --- |
| William’s E Medium | Basal medium | Gibco | 12551-032 |
| L-glutamine | 2 mM | Biowest | X0550 |
| Insulin | 5 µg/mL | PAN-Biotech | P07-04300 |
| Hydrocortisone 21-hemisuccinate sodium salt | 50 µM | Sigma-Aldrich | H2270-100MG |
| Penicillin/streptomycin | 1× | Biowest | L0022 |
| Insulin-transferrin-selenium (ITS) | 1× | Gibco | 41400045 |
| Sodium pyruvate | 1 mM | Sigma-Aldrich | S8636-100ML |
| 2-Mercaptoethanol | 55 µM | Gibco | 21985-023 |
| Recombinant human fibronectin | 1.5 µg/mL | R&D Systems | 1918-FN |
| Dimethyl sulfoxide (DMSO) | 1% for 24 h, then 1.7% for 14 days | Sigma-Aldrich | D2650 |

**Table S2**. Composition of the chemically defined media used for HuH7 cells.

| **Component** | **Final concentration** | **Supplier** | **Catalog number** |
| --- | --- | --- | --- |
| DMEM/F12 | Basal medium | Biowest | L0090 |
| CD lipid concentrate | 1:100 | Gibco | 11905-031 |
| MEM non-essential amino acids (NEAA) | 1× | Biowest | X0557-100 |
| HEPES | 10 mM | Biowest | L0180 |
| Pluronic F-68™ | 0.1% | Applichem | A1288 |
| GlutaMAX™ | 2 mM | Gibco | 35050 |
| D-(+)-glucose | 4.5 g/L | Sigma-Aldrich | G8769 |
| Penicillin/streptomycin | 1× | Biowest | L0022 |
| Insulin-transferrin-selenium (ITS) | 1× | Gibco | 41400045 |
| Recombinant human epidermal growth factor (EGF) | 10 ng/mL | Gibco | PHG0313 |
| Recombinant human hepatocyte growth factor (HGF) | 10 ng/mL | Gibco | PHG0254 |
| Human placental collagen type I | 32 µg/mL | THT Biomaterials GmbH | THT0102 |

**Table S3**. ***Table_S1_detox_panels_similarity_and_matrices.xlsx****. Detox panel benchmarking. Detox gene-panel expression matrices and PHH similarity scores (Phase I/II and transporters).*

**Table S4**. ***Table_S2_identity_panels_and_PHH_similarity.xlsx****. Identity panel benchmarking. PHH-referenced similarity scores for hepatic identity gene panels across HepaRG and HuH7 in FBS-SM vs CDM.*

***Table S5****. Sequencing yield and primary read quality metrics per sample.*

| ID | Sample name | Total raw reads | Total non-rRNA reads | Total HQ reads | HQ bases (Q30) | GC content | rRNA reads (%) | HQ reads (%) |
| --- | --- | --- | --- | --- | --- | --- | --- | --- |
| 1 | HuH7_CDM_1 | 66 M | 64.67 M | 63.96 M | 92.53% | 48.87% | 2.02% | 96.91% |
| 2 | HuH7_CDM_2 | 66 M | 65.65 M | 64.85 M | 96.10% | 49.04% | 0.54% | 98.25% |
| 3 | HuH7_CDM_3 | 66 M | 65.58 M | 64.84 M | 92.90% | 48.89% | 0.63% | 98.24% |
| 4 | HuH7_FBS-SM_1 | 66 M | 65.56 M | 64.81 M | 92.71% | 49.02% | 0.66% | 98.19% |
| 5 | HuH7_FBS-SM_2 | 66 M | 65.59 M | 64.77 M | 92.82% | 49.08% | 0.62% | 98.14% |
| 6 | HuH7_FBS-SM_3 | 66 M | 65.44 M | 64.70 M | 92.61% | 49.12% | 0.84% | 98.02% |
| 7 | PHH_1 | 66 M | 64.09 M | 63.34 M | 96.50% | 49.00% | 2.89% | 95.97% |
| 8 | PHH_2 | 66 M | 63.79 M | 63.05 M | 96.93% | 49.52% | 3.35% | 95.53% |
| 9 | HepaRG_CDM_1 | 66 M | 65.24 M | 64.54 M | 92.94% | 48.68% | 1.14% | 97.79% |
| 10 | HepaRG_CDM_2 | 66 M | 65.16 M | 64.41 M | 92.91% | 48.67% | 1.27% | 97.59% |
| 11 | HepaRG_CDM_3 | 66 M | 65.25 M | 64.54 M | 92.94% | 48.73% | 1.14% | 97.79% |
| 12 | HepaRG_FBS-SM_1 | 66 M | 65.04 M | 64.29 M | 92.24% | 49.88% | 1.46% | 97.40% |
| 13 | HepaRG_FBS-SM_2 | 66 M | 65.25 M | 64.47 M | 95.63% | 48.72% | 1.14% | 97.68% |
| 14 | HepaRG_FBS-SM_3 | 66 M | 65.25 M | 64.52 M | 92.79% | 48.60% | 1.14% | 97.76% |
| 15 | Hela_CDM_1 | 76 M | 75.55 M | 73.68 M | 95.92% | 49.73% | 0.59% | 96.95% |
| 16 | Hela_CDM_2 | 76 M | 75.54 M | 73.26 M | 95.93% | 49.79% | 0.61% | 96.40% |
| 17 | Hela_CDM_3 | 68.94 M | 68.76 M | 67.14 M | 97.09% | 49.70% | 0.26% | 97.40% |
| 18 | Hela_FBS-SM_1 | 75.46 M | 74.92 M | 73.01 M | 96.01% | 49.58% | 0.73% | 96.75% |
| 19 | Hela_FBS-SM_2 | 76 M | 75.56 M | 73.51 M | 96.01% | 49.75% | 0.58% | 96.72% |
| 20 | Hela_FBS-SM_3 | 74.55 M | 74.20 M | 72.82 M | 95.88% | 49.73% | 0.47% | 97.67% |

***Table S6****. Alignment and mapping metrics per sample.*

| ID | Sample | Total HQ reads | Mapped reads | Unmapped reads | Unique reads |
| --- | --- | --- | --- | --- | --- |
| 1 | HuH7_CDM_1 | 63.96 M | 63.43 M (99.17%) | 532.99 K (0.83%) | 62.33 M (97.45%) |
| 2 | HuH7_CDM_2 | 64.85 M | 64.23 M (99.05%) | 617.23 K (0.95%) | 63.07 M (97.26%) |
| 3 | HuH7_CDM_3 | 64.84 M | 64.34 M (99.24%) | 494.14 K (0.76%) | 63.12 M (97.36%) |
| 4 | HuH7_FBS-SM_1 | 64.81 M | 64.31 M (99.24%) | 494.68 K (0.76%) | 63.19 M (97.50%) |
| 5 | HuH7_FBS-SM_2 | 64.77 M | 64.27 M (99.22%) | 502.99 K (0.78%) | 63.06 M (97.36%) |
| 6 | HuH7_FBS-SM_3 | 64.70 M | 64.20 M (99.23%) | 498.86 K (0.77%) | 63.08 M (97.50%) |
| 7 | PHH_1 | 63.34 M | 61.86 M (97.67%) | 1.48 M (2.33%) | 58.94 M (93.06%) |
| 8 | PHH_2 | 63.05 M | 61.28 M (97.19%) | 1.77 M (2.81%) | 58.53 M (92.83%) |
| 9 | HepaRG_CDM_1 | 64.54 M | 64.00 M (99.16%) | 541.54 K (0.84%) | 62.52 M (96.87%) |
| 10 | HepaRG_CDM_2 | 64.41 M | 63.85 M (99.12%) | 565.48 K (0.88%) | 62.37 M (96.84%) |
| 11 | HepaRG_CDM_3 | 64.54 M | 63.99 M (99.15%) | 549.70 K (0.85%) | 62.46 M (96.78%) |
| 12 | HepaRG_FBS-SM_1 | 64.29 M | 63.71 M (99.10%) | 577.76 K (0.90%) | 62.32 M (96.94%) |
| 13 | HepaRG_FBS-SM_2 | 64.47 M | 63.83 M (99.02%) | 634.43 K (0.98%) | 62.34 M (96.70%) |
| 14 | HepaRG_FBS-SM_3 | 64.52 M | 63.95 M (99.11%) | 577.35 K (0.89%) | 62.52 M (96.90%) |
| 15 | Hela_CDM_1 | 73.68 M | 72.88 M (98.91%) | 803.44 K (1.09%) | 71.43 M (96.94%) |
| 16 | Hela_CDM_2 | 73.26 M | 72.41 M (98.84%) | 850.05 K (1.16%) | 71.02 M (96.94%) |
| 17 | Hela_CDM_3 | 67.14 M | 66.56 M (99.13%) | 586.98 K (0.87%) | 65.18 M (97.08%) |
| 18 | Hela_FBS-SM_1 | 73.01 M | 72.11 M (98.76%) | 906.93 K (1.24%) | 70.43 M (96.46%) |
| 19 | Hela_ FBS-SM_2 | 73.51 M | 72.65 M (98.84%) | 854.40 K (1.16%) | 70.89 M (96.44%) |
| 20 | Hela_FBS-SM_3 | 72.82 M | 71.44 M (98.11%) | 1.37 M (1.89%) | 69.83 M (95.90%) |

***Table S7****. Distribution of aligned reads across genomic features per sample.*

| ID | Sample name | exonic | intronic | intergenic | overlapping exon |
| --- | --- | --- | --- | --- | --- |
| 1 | HuH7_CDM_1 | 53,821,399 (90.17%) | 4,952,447 (8.3%) | 912,317 (1.53%) | 4,240,933 (7.11%) |
| 2 | HuH7_CDM_2 | 54,641,477 (90.48%) | 4,828,455 (8%) | 921,793 (1.53%) | 4,151,775 (6.87%) |
| 3 | HuH7_CDM_3 | 54,554,753 (90.22%) | 4,960,055 (8.2%) | 951,886 (1.57%) | 4,151,589 (6.87%) |
| 4 | HuH7_FBS-SM_1 | 54,706,606 (90.27%) | 5,015,947 (8.28%) | 879,585 (1.45%) | 4,293,205 (7.08%) |
| 5 | HuH7_FBS-SM_2 | 54,499,133 (90.28%) | 4,951,014 (8.2%) | 913,982 (1.51%) | 4,163,444 (6.9%) |
| 6 | HuH7_FBS-SM_3 | 54,490,965 (90.13%) | 5,066,622 (8.38%) | 902,161 (1.49%) | 4,291,037 (7.1%) |
| 7 | PHH_1 | 39,789,644 (87.43%) | 5,198,774 (11.42%) | 520,964 (1.14%) | 3,172,283 (6.97%) |
| 8 | PHH_2 | 40,687,603 (87.57%) | 5,255,539 (11.31%) | 518,573 (1.12%) | 3,261,425 (7.02%) |
| 9 | HepaRG_CDM_1 | 51,475,677 (90.19%) | 4,564,956 (8%) | 1,037,088 (1.82%) | 3,892,144 (6.82%) |
| 10 | HepaRG_CDM_2 | 51,236,611 (89.63%) | 4,824,769 (8.44%) | 1,101,735 (1.93%) | 3,906,622 (6.83%) |
| 11 | HepaRG_CDM_3 | 50,992,066 (89.88%) | 4,686,019 (8.26%) | 1,054,958 (1.86%) | 3,915,508 (6.9%) |
| 12 | HepaRG_FBS-SM_1 | 53,662,965 (90.39%) | 4,816,590 (8.11%) | 890,506 (1.5%) | 4,274,275 (7.2%) |
| 13 | HepaRG_FBS-SM_2 | 52,085,987 (90.04%) | 4,710,439 (8.14%) | 1,053,212 (1.82%) | 3,857,095 (6.67%) |
| 14 | HepaRG_FBS-SM_3 | 53,223,475 (90.42%) | 4,613,366 (7.84%) | 1,028,102 (1.75%) | 3,991,822 (6.78%) |
| 15 | Hela_CDM_1 | 60,795,068 (89.34%) | 6,526,743 (9.59%) | 724,360 (1.06%) | 4,782,472 (7.03%) |
| 16 | Hela_CDM_2 | 61,047,378 (90.25%) | 5,912,007 (8.74%) | 684,496 (1.01%) | 4,773,254 (7.06%) |
| 17 | Hela_CDM_3 | 55,814,633 (90.05%) | 5,531,112 (8.92%) | 637,680 (1.03%) | 4,386,690 (7.08%) |
| 18 | Hela_FBS-SM_1 | 61,881,496 (92.37%) | 4,413,817 (6.59%) | 700,239 (1.05%) | 4,524,895 (6.75%) |
| 19 | Hela_FBS-SM_2 | 62,105,513 (92.38%) | 4,411,033 (6.56%) | 712,770 (1.06%) | 4,495,801 (6.69%) |
| 20 | Hela_FBS-SM_3 | 60,203,596 (91.73%) | 4,695,051 (7.15%) | 734,978 (1.12%) | 4,410,376 (6.72%) |

**Table S8**. ***Table_S8_DESeq2_differential_expression_outputs.xlsx****. DESeq2 outputs. Full gene-level DESeq2 results.*

**Table S9**. ***Table_S9_QuickGO_ssGSEA_outputs.xlsx****. QuickGO ssGSEA scores. Per-sample ssGSEA matrices (GSVA/ssGSEA on DESeq2 VST) and condition summaries for the GO:BP term sets.*

**Table S10**. ***Table_S10_GO_BP_enrichment_outputs.xlsx****. GO:BP enrichment results. Complete clusterProfiler GO:BP over-representation outputs.*

***Fig. S1*** *Sample-level transcriptomic distance structure. Sample-by-sample Euclidean distance heatmap computed from DESeq2 VST expression values, with annotation tracks indicating cell model and medium (CDM, FBS-SM, or PHH reference).*

**Fig. S2** Venn diagram of direction-resolved DEG overlaps between HepaRG and HuH7 under CDM versus FBS-SM. The four sets represent genes upregulated or downregulated in CDM relative to FBS-SM in HepaRG and HuH7 cells, using DESeq2 significance criteria of FDR < 0.05 and |log2FC| > 0.5.

**Fig. S3** HepaRG benchmark differential expression relative to PHH. Full-size volcano plots showing differential expression of HepaRG cultured in (a) CDM and (b) FBS-SM relative to pooled PHH. Axes and thresholds match the main DESeq2 criteria (FDR < 0.05 and |log2FC| > 0.5), with representative high-effect and/or highly significant genes annotated.

***Fig. S4*** *HuH7 benchmark differential expression relative to PHH. Full-size volcano plots showing differential expression of HuH7 cultured in (a) CDM and (b) FBS-SM relative to pooled PHH.* Axes and thresholds match the main DESeq2 criteria (FDR < 0.05 and |log2FC| > 0.5), with representative high-effect and/or highly significant genes annotated.

**Fig. S5** Direction-resolved GO:BP enrichment for HepaRG CDM versus FBS-SM. Simplified GO:BP enrichment dotplots for HepaRG genes (a) upregulated and (b) downregulated in CDM versus FBS-SM. Dot size denotes GeneRatio and dot color denotes adjusted P value.

***Fig. S6*** *Direction-resolved GO:BP enrichment for HuH7 CDM versus FBS-SM. Simplified GO:BP enrichment dotplots for HuH7 genes (a) upregulated and (b) downregulated in CDM versus FBS-SM. Dot size denotes GeneRatio and dot color denotes adjusted P value.*

***Fig. S7*** *Direction-resolved GO:BP enrichment for HepaRG in CDM benchmarking versus PHH. Simplified GO:BP enrichment dotplots for HepaRG differential expression (a) upregulated and (b) downregulated relative to PHH (direction-resolved).*

***Fig. S8*** *Direction-resolved GO:BP enrichment for HuH7 in CDM benchmarking versus PHH. Simplified GO:BP enrichment dotplots for HuH7 differential expression (a) upregulated and (b) downregulated relative to PHH (direction-resolved).*

***Fig. S9*** *PHH-referenced similarity scores for hepatic identity marker categories. Quantitative similarity summaries benchmarking HepaRG and HuH7 (CDM and FBS-SM) to PHH across hepatic identity marker categories (adult hepatocyte, fetal/progenitor, inflammation/stress). Similarity scores were computed from gene-set expression deviations relative to PHH using an RMSE-based normalization (PHH fixed at 1).*

***Fig. S10*** *Full resolution ssGSEA pathway profiling benchmarked to PHH. High-resolution heatmap of ssGSEA scores (z-scored) computed from DESeq2 VST expression values for QuickGO-derived GO:BP term sets,* ***provided as a separate pdf file (Fig_S10_PHH_benchmark_pathways.pdf)****.*

***Figure S11. Targeted qPCR validation of cytochrome P450 expression and rifampicin responsiveness.*** *Expression of CYP1A2, CYP2C9, CYP3A4, and CYP7A1 in HuH7 and differentiated HepaRG cells after treatment with rifampicin for 72 h, quantified by qPCR and normalized to GAPDH. (a) Absolute normalized expression was calculated as* $2^{-\Delta Ct}$*. (b) CDM/FBS-SM ratios were calculated from absolute normalized expression values. (c) Rifampicin responses were calculated as* $2^{-\Delta\Delta Ct}$*relative to matched untreated controls.*

Nessar A, Röhrs V, Ziersch M, et al. (2025) Promoting ethical and reproducible cell culture: implementing animal-free alternatives to teaching in molecular and cell biology. Volume 7 - 2025 doi:10.3389/ftox.2025.1670513
